# Localized bullous pemphigoid: Four clinical cases and a literature review

**DOI:** 10.1002/ccr3.2697

**Published:** 2020-02-11

**Authors:** Chloe Algoet, Sabine Mostinckx, Ivan Theate, Olivier Vanhooteghem

**Affiliations:** ^1^ Dermatology Unit CHU UCL Namur–Sainte Elisabeth Hospital Namur Belgium; ^2^ Institute of Pathology and Genetics Gosselies Belgium

**Keywords:** autoimmune disease, comorbidities, localized bullous pemphigoid, pemphigoid

## Abstract

Localized bullous pemphigoid (LBP) rarely evolves into the generalized form, and the prognosis is better. In our opinion, the occurrence of LBP is underestimated because of incorrect diagnoses. It is therefore important to perform a skin biopsy each time a bullous rash is concerned in order to make a definite diagnosis.

## Graphical abstract

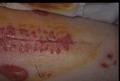

## 1 INTRODUCTION

Localized bullous pemphigoid (LBP) is a specific form of bullous pemphigoid. It can appear on any wound site. Here, we review the circumstances contributing to the appearance of LBP, the predisposing factors of LBP and the evolution and treatment of LBP in 4 patients. A literature review leads to a better understanding of the appearance of LBP.

Localized bullous pemphigoid (LBP) is a specific form of bullous pemphigoid. It can appear on any wound or surgery site; after radiotherapy, PUVA therapy or dynamic phototherapy; in patients with chronic edema of the lower limbs; or in patients suffering from metastatic melanoma who are treated or not treated with anti‐PD‐1. LBP diagnosis should not be overlooked; it should be diagnosed by a skin biopsy, prevented and monitored to avoid spreading.

## 2 CLINICAL CASES

### 2.1 Case 1

**Figure 1:**
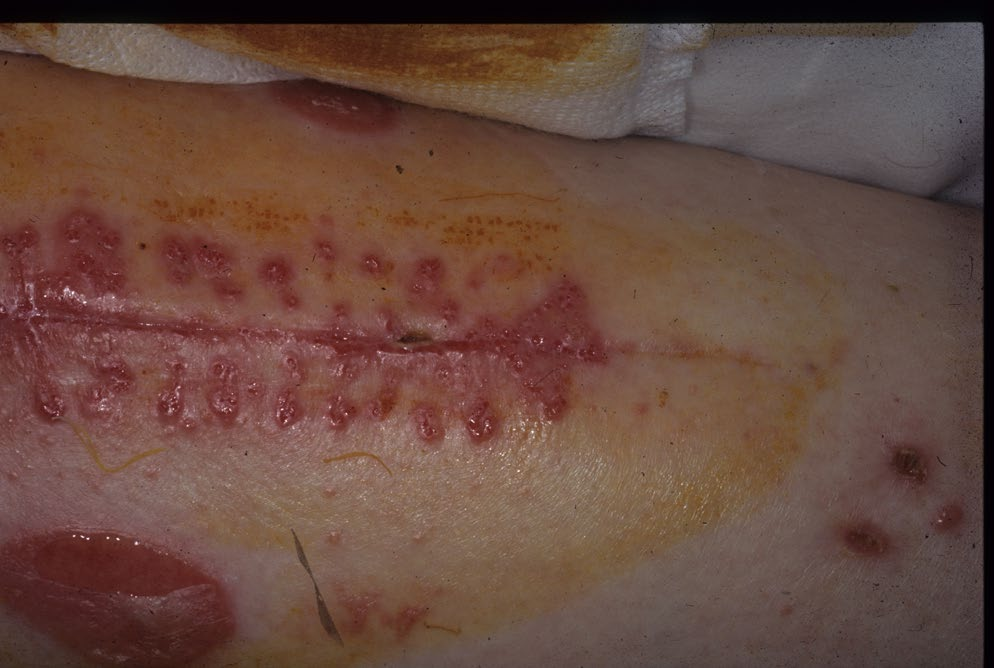
Erythemal and phlyctenular scar onto the areas surrounding the surgical wound

A 78‐year‐old woman without any major medical history is operated on to replace a hip as a result of arthrosis. The patient does not take any medication. Ten days after the operation, the patient develops a light but widespread pruritus that is more developed around the site of the surgery but not the result of a particular lesion. An irritated dermatitis is diagnosed and treated with emollients. In the following days, in addition to the pruritus symptomatology, the scar and the areas surrounding the surgical wound and suture threads become erythemal and phlyctenular (Figure 1). No skin or mucosal lesions can be observed. A blood test shows a slight inflammatory response.

### 2.2 Case 2

**Figure 2:**
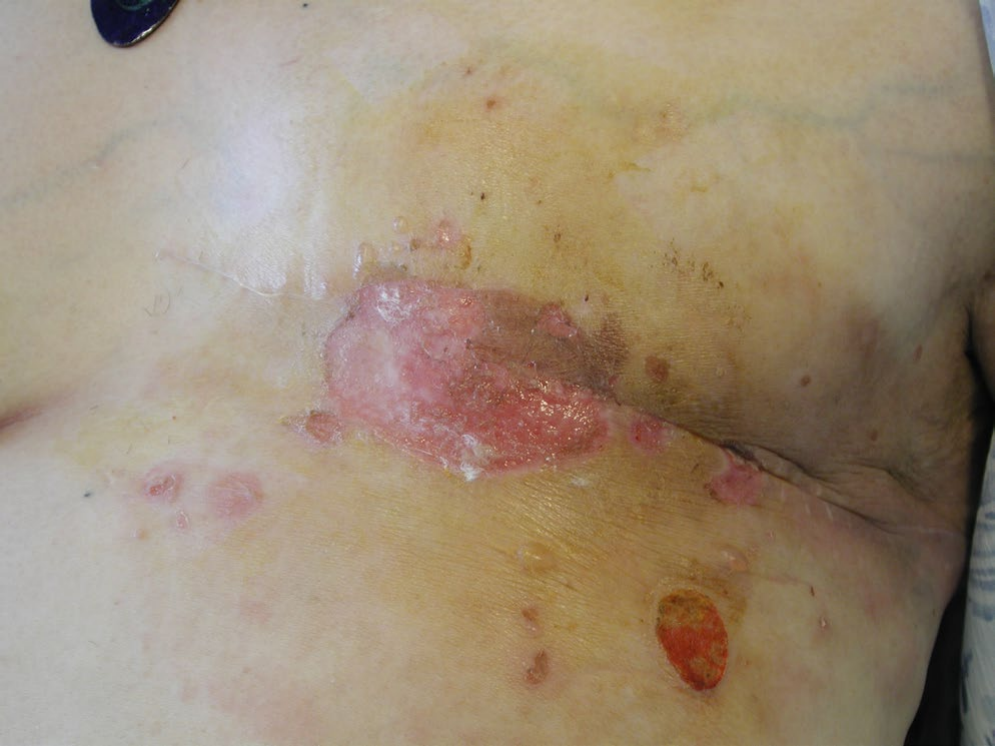
Bullous rash localized on the mammectomy scar

Ten years ago, a 70‐year‐old woman suffered from neoplasia and underwent a left mammectomy and radiotherapy. She presents with a bullous rash localized on the scar (Figure 2). The patient does not take any medication. She is regularly watched in oncology, and her laboratory workup is satisfactory.

The lesions have been present for 6 months and have no obvious cause. They are accompanied by uncontrollable localized pruritus.

The clinical examination highlights phlyctenular pruritus on erythemal background evolving toward skin erosion. The lesions are limited to the mammectomy area. Skin and mucosa are otherwise within normal limits.

### 2.3 Case 3

**Figure 3:**
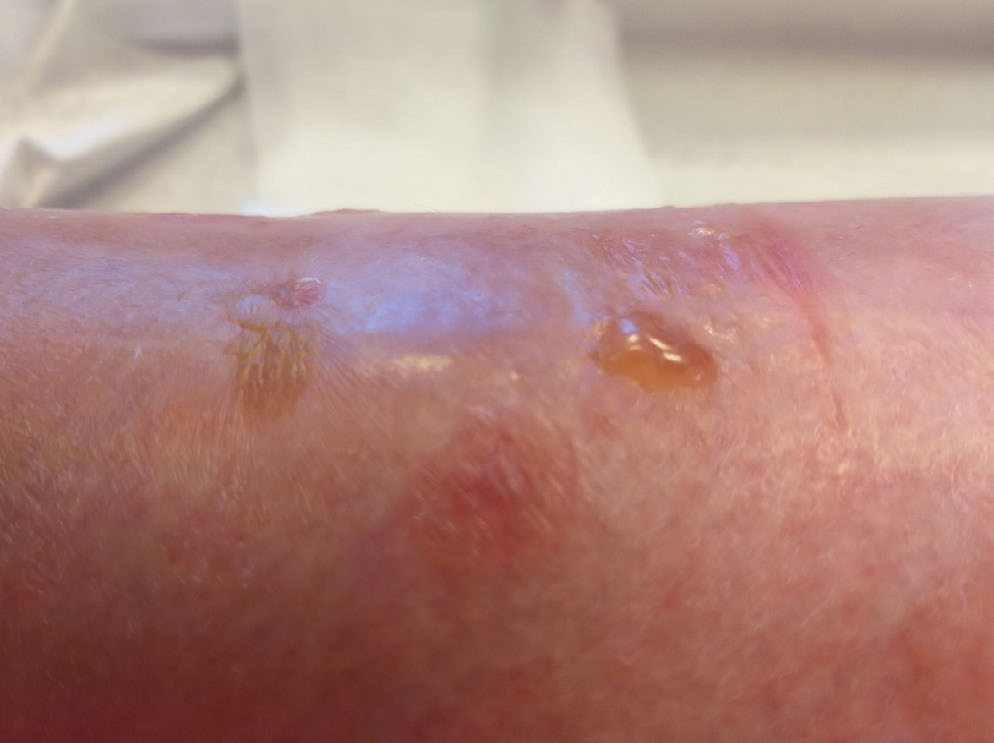
Bullous tense lesions on one side of the left tibial crest

An 82‐year‐old woman without major medical history suffers from an oozing and pruritic erythemal patch on one side of the left tibial crest. Bullous tense lesions appear on the plaque during the following weeks (Figure 3). The patient does not suffer from edema of the lower limbs, and no specific severe trauma has been reported. The patient has not undergone radiotherapy on the site and does not take any specific medication. Bulla appears only around the area of the sock elastic band. Skin and mucosa are otherwise within normal limits.

### 2.4 Case 4

**Figure 4:**
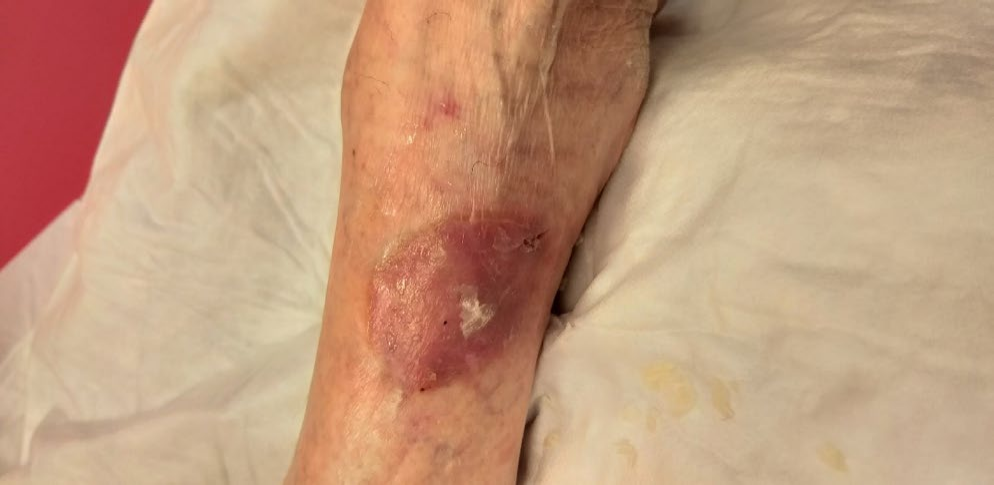
Phlyctenular lesion on the left ankle

An 87‐year‐old woman suffering from Alzheimer's disease has been developing a unilateral erosive, purplish patch on the left ankle for some weeks. The plaque is highly painful but only a little pruritic and becomes phlyctenular within 3 weeks (Figure 4). Skin and mucosa are otherwise within normal limits. The patient does not take any specific medication.

The histology of these four cases shows a partially detached epidermis with a subepidermal bulla. There is a slight inflammatory reaction of the dermis, mainly composed of lymphocytes and some neutrophilic and eosinophilic granulocytes. There is no vasculitis. Direct immunofluorescence shows immunoglobulins G and C3 in the dermo‐epidermal boundary. Thanks to clinical pictures, histology, and immunofluorescence, localized bullous pemphigoid is diagnosed. Patients are treated locally with powerful corticoids (clobetasol propionate 0.05%—ointment) twice a day for 10 days with complete healing of the lesions. In the first two cases, there was no recurrence or any other localization of the illness for 26 months after diagnosis and treatment. The two other patients were lesion‐free for 6 months, without any recurrence or any other localization.

## 3 DISCUSSION

Bullous pemphigoid (PB) is the most frequent autoimmune bullous pemphigoid. It is characterized by the production of antibodies against BP180 and BP230 proteins, which are normal components of hemidesmosomes found in the dermo‐epidermal boundary.^1^ The binding of antibodies leads to complementary activation, the recruitment of inflammatory cells, and the release of proteolytic enzymes.^2^ BP is mostly generalized, but in some rare cases, it can be localized (LBP). It can remain localized or, rarely, precede generalization.^3^ LBP either appears on trauma sites (ie, surgery scars due to amputation) or colostomy sites,^4^ in which case it is called LBP of “Brunsting and Perry” as described in 1957, or LBP predominates in the lower limbs without being associated with trauma.^5^ When it occurs at a trauma site and in cases of secondary spreading, a concentration of the symptoms can be observed at the trauma site.^6^ Some authors mention the creation or appearance of neo‐antigens by the trauma, while others believe that the anti‐BP180 and anti‐BP230 antibodies are already present and that trauma would recruit those antibodies.^7^ “Koebnerization” is defined as the appearance of new lesions after trauma in patients suffering from a skin disease. The illness can preexist or appear after a trauma. In cases of LBP appearing after burns or surgical scars, it is easy to conceive that the trauma acts as a contributing factor. Surgeries or accidental traumas, as well as their resulting scars, modify lymph circulation and could altogether provoke an immune‐neural lack of balance in the traumatized area, which would damage those areas and make them immunologically reactive. In those vulnerable sites, LBP can appear after a variable period of some days to several decades.^8^ Moreover, the localized traumas that can provoke LBP development may also come in various forms and be insignificant, such as the pressure of a sock elastic band.^5^ LBP can also appear on radiotherapy sites.^9^ According to a study by Nguyen and Coll, LBP develops in 72% of cases within six months after the end of radiotherapy and in 28% of cases during radiotherapy.^10^ The appearance of LBP on the mouth after radiotherapy against laryngeal carcinoma has also been described.^11^ The pathogenic mechanism of LBP development associated with radiotherapy is difficult to prove and remains hypothetical. The antigens responsible for the illness are freed by epidermal cells, possibly by tumor cells. They are then ingested by immature Langerhans cells, treated and presented to major histocompatibility class II molecules. These antigen‐presenting cells present the antigen(s) (BP180 and/or BP230) to T‐cell receptors. The activated T cells then present it/them to the B cells, which selectively produce antibodies against the “BP” antigen. Fixing of the antibodies against BP180 and BP230 activates the complementary system. Simultaneously, the binding of anti‐BP auto‐antibodies provokes the secretion of IL‐6 and IL‐8 by basal keratinocytes, which attracts neutrophils and eosinophils. Metalloproteinase‐9 and elastase derived from neutrophils cleave the intracellular domain of BP180 and ease the separation of the dermis from the epidermis.

The presence of LBP is also reported in the lower limbs of patients with chronic vein insufficiency.^12^ Physical and immunological modifications associated with chronic vein stasis can predispose patients to the presentation of the antigens that are responsible for the illness, to the auto‐reactive T cells and to the subsequent B cells producing auto‐antibodies. Moreover, vein stasis provokes an extravasation of blood and plasma constituents, including the mediators of innate and adaptative immunity, in the surrounding tissues.^12^, ^13^ LBP can also appear after treatment with PUVA therapy, UVB therapy, or dynamic phototherapy.^14^ The appearance mechanism of LBP can be identical to the after‐burn mechanism. Lastly, many cases of LBP have been reported among patients suffering from metastatic melanomas, treated or not treated with pembrolizumab (anti‐PD‐1). Inhibition of PD‐1/PD‐L1 pathways by anti‐PD‐1s leads to a decrease in immunological tolerance toward BP180 antigens. This drives the development of an immunological response mainly against BP180 antigen, which may be overexpressed at the surface of malignant melanocytes. Patients suffering from melanoma and concomitantly receiving anti‐PD‐1s might be overexposed to BP180 antigens, which would lead to T‐cell dysregulation altogether with the production of antibodies specific to BP180 antigens.^15^ Naidoo et al suggest the coexistence of a humoral response via stimulation of the germinal center of B cells by PD‐1+ follicular T‐helper cells,^16^ and Hirotsu et al speculate that a treatment combining radiotherapy and a PD‐1 inhibitor could potentiate the risk of developing LBP during treatment.^17^ Many predisposing factors for the development of BP and LBP are generally common to both variants; however, many studies show an association between HLA‐DQb1*0301 and specific clinical variants of BP.^2^ Patients carrying the HLA‐DQb1*0301 allele might present an increased need for T lymphocytes for many epitopes of BP180, particularly in the field of BP180‐NC16a. Therefore, these patients would have an increased genetic sensitivity toward developing LBP during exposure to the target antigene.^18^ In addition, patients suffering from an underlying neurologic disease might be exposed to predetermined sequestered auto‐antigens, mainly BP180. They are henceforth also more likely to develop BP‐type illnesses. Old age or specific medications (neuroleptics, spironolactones, loop diuretics, etc) are also mentioned as risk factors for developing LBP or BP.^19^ Differential diagnosis is essential for contact dermatitis, irritative dermatitis, other bullous illnesses, and viral infections, such as the varicella‐zoster virus or herpes. A diagnosis is unequivocal only in association with a clinical examination, with histology and with immunofluorescence.^6^ LBP is preferably treated with the application of powerful topical steroids to the damaged skin, with a progressive reduction of the application only after 15 days. In the case of resistance to topical treatments, it was shown that simultaneous administration of 0.5 mg/kg/day of prednisolone is effective. Then, systemic doses must be progressively reduced to reach minimal therapy (prednisone 0.1 mg/kg/day) within 4‐6 months after beginning the treatment. Although the optimal duration remains uncertain, the total recommended treatment time (consolidation and maintenance phases) is 4‐12 months. The use of an immunosuppressant or immunomodulating treatment sparing the use of corticoids must be considered in cases of contraindication to oral corticosteroids or in cases of severe comorbidity (ie, diabetes, severe osteoporosis, and severe cardiovascular disorders).^20^

In conclusion, the prognosis of LBP is better than that of BP because it responds better to local treatments than the generalized form.^21^ Moreover, it rarely evolves into the generalized form. In our opinion, the occurrence of LBP is underestimated because of incorrect diagnoses. It is therefore important to perform a skin biopsy each time a bullous rash is concerned in order to make a definite diagnosis and to prevent any adverse outcome of the illness. It is also necessary to highlight the cases reported in clinical practice to develop a better defined set of risk factors and to improve prevention and early prognosis of the illness.

## CONFLICT OF INTEREST

None declared.

## AUTHOR CONTRIBUTIONS

All authors have contributed at this work.
